# Improvement in adenoma detection rate by artificial intelligence-assisted colonoscopy: Multicenter quasi-randomized controlled trial

**DOI:** 10.1055/a-2521-5169

**Published:** 2025-02-26

**Authors:** Ronja Maria Birgitta Lagström, Karoline Bendix Bräuner, Julia Bielik, Andreas Weinberger Rosen, Julie Gräs Crone, Ismail Gögenur, Mustafa Bulut

**Affiliations:** 1 524788 Department of Surgery, Zealand University Hospital Koge, Køge, Denmark; 2 73014 Department of Surgery, Slagelse Hospital, Slagelse, Denmark; 3 53163 Department of Surgery, Holbæk Sygehus, Holbæk, Denmark; 4 91907 Department of Surgery, Næstved Hospital, Næstved, Denmark; 5 53139 Department of Clinical Medicine, University of Copenhagen Faculty of Health and Medical Sciences, Copenhagen, Denmark; 6 Copenhagen Academy for Medical Education and Simulation (CAMES), Copenhagen, Denmark

**Keywords:** Endoscopy Lower GI Tract, Polyps / adenomas / ..., CRC screening, Endoscopic resection (polypectomy, ESD, EMRc, ...), Colorectal cancer

## Abstract

**Background and study aims:**

Adenoma detection rate (ADR) is a key performance measure with variability among endoscopists. Artificial intelligence (AI) in colonoscopy could reduce this variability and has shown to improve ADR. This study assessed the impact of AI on ADR among Danish endoscopists of varying experience levels.

**Patients and methods:**

We conducted a prospective, quasi-randomized, controlled, multicenter trial involving patients aged 18 and older undergoing screening, surveillance, and diagnostic colonoscopy at four centers. Participants were assigned to AI-assisted colonoscopy (GI Genius, Medtronic) or conventional colonoscopy. Endoscopists were classified as experts (> 1000 colonoscopies) or non-experts (≤ 1000 colonoscopies). The primary outcome was ADR. We performed a subgroup analysis stratified on endoscopist experience and a subset analysis of the screening population.

**Results:**

A total of 795 patients were analyzed: 400 in the AI group and 395 in the control group. The AI group demonstrated a significantly higher ADR than the control group (59.1% vs. 46.6%, *P* < 0.001). The increase was significant among experts (59.9% vs. 47.3%, *P* < 0.002) but not among non-experts. AI assistance significantly improved ADR (74.4% vs. 58.1%, *P* = 0.003) in screening colonoscopies. Polyp detection rate (PDR) was also higher in the AI group (69.8% vs. 56.2%, *P* < 0.001). There was no significant difference in the non-neoplastic resection rate (NNRR) (15.1% vs. 17.1%, *P* = 0.542).

**Conclusions:**

AI-assisted colonoscopy significantly increased ADR by 12.5% overall, with a notable 16.3% increase in the screening population. The unchanged NNRR indicates that the higher PDR was due to increased ADR, not unnecessary resections.

## Introduction

Colorectal cancer (CRC) is the third most frequent cancer and the second most common cause of cancer-related death worldwide 1 . Screening colonoscopy with removal of precancerous lesions is associated with a reduced risk of CRC-related death 2 . Adenoma detection rate (ADR) is the percentage of colonoscopies in which one or more adenomas are found. It is inversely correlated with incidence of interval CRC, which means cancer occurring in the interval between scheduled colonoscopies. Unfortunately, adenomas are frequently missed 3 and ADR varies widely among different endoscopists 4 .

Artificial intelligence (AI) in colonoscopy can reduce performance variability and compensate for perceptual errors by aiding polyp detection on colonoscopy images in real time 5 .

In the last few years, several randomized controlled trials (RCT) have been published showing that use of AI contributes to a significantly higher ADR, compared with colonoscopies without AI assistance 6 7 8 9 10 11 12 13 .

However, because referral for colonoscopies varies greatly from country to country, it can be difficult to translate results from the current literature to clinical practice in Northern Europe. Approximately one-third of RCTs on the effect of AI assistance on ADR in the current literature originate from China, where baseline ADR is not comparable to that of Western countries 6 8 10 11 , and we anticipate that the indication for colonoscopy and compliance with participation may differ across countries.

To the best of our knowledge, there are no published Northern European studies investigating the effect of AI-assisted colonoscopy in a sufficiently large cohort in a multicenter setting.

We evaluated the effect of a computer-aided detection system (CADe) on ADR at four different centers in a Danish population. We aimed to investigate whether introduction of AI assistance would improve ADR. We also wanted to examine whether ADR was affected by endoscopist experience, the endoscopy being for screening, or time of day (before and after noon) when endoscopy was performed.

## Patients and methods

We performed a prospective, multicenter, non-blinded, quasi-randomized, controlled crossover trial. The trial was registered at “The Region Zealand record of processing activities related to scientific research projects” (REG-092–2022) 23.09.2022, and at ClinicalTrials.gov (NCT05740137).

### Eligibility

Patients were 18 years or older, referred either for screening due to a positive fecal immunochemical test (FIT) (> 100 μg/L), surveillance, or diagnostic colonoscopy at four centers in Region Zealand, Denmark. We excluded patients referred for removal of previously detected polyps, patients referred for control colonoscopies due to inflammatory bowel disease, and emergency colonoscopies. Subjects where the bowel preparation was so poor that it prevented the colonoscopy from being performed were excluded from the analysis. If cancer was suspected during the colonoscopy, the patient was excluded. This was a quality development study testing a marketed product as a standard of care, and thus, there was no additional need for patient consent, other than the consent to perform the procedure, nor was approval needed from the Danish Research Ethics Committees.

### AI system

**Fig. 1:**
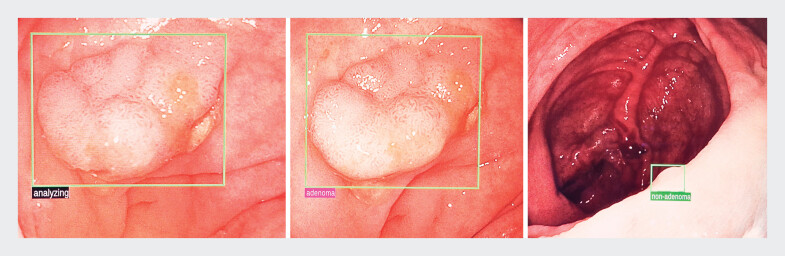
Colonic lesions analyzed by GI Genius (Medtronic) are characterized as adenoma (second image) or non-adenoma (third image).

We used the AI system GI Genius (generation 3, Medtronic), a combined computer-aided detection (CADe) and characterization system (CADx). It is an adjunct to standard video colonoscopy, trained to process colonoscopy images in real time. The system highlights lesions suspected to have visual characteristics consistent with different types of mucosal abnormalities. Detected lesions are simultaneously marked on the screen and characterized as “adenoma,” “non-adenoma,” or “no prediction” ( Fig. 1 ). The system´s CADe function has previous been tested in other randomized trials 14 15 16 .

### Randomization

**Fig. 2:**
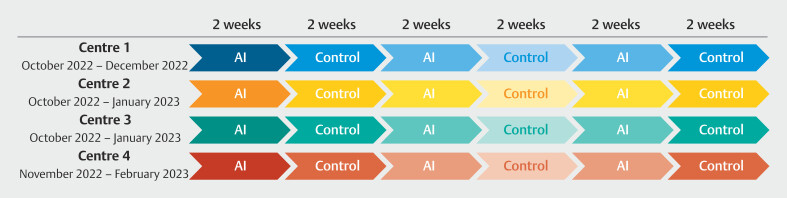
Quasi-randomization at center level.

Randomization was done at a center level using a quasi-randomization approach. The centers used rapid cycles of 2 weeks of practice change, meaning that there was 2 weeks using AI followed by 2 weeks of using the current standard procedure without AI. These cycles were repeated until all patients were included. Centers started inclusion with approximately 1 week of staggering ( Fig. 2 ). Because there was no change in rules for referral and indication for colonoscopy between AI and non-AI weeks, there was assumed to be no difference between patients included in AI and non-AI weeks.

### Colonoscopy procedures

We included expert endoscopists (> 1000 colonoscopies performed prior to the trial) and non-experts (≤ 1000 colonoscopies). Bowel preparation and sedation were given according to regional guidelines, and they varied among patients, depending on their individual needs and restrictions. Standard preparation for screening colonoscopies was Moviprep (polyethylene glycol 3350, sodium sulfate, sodium chloride, potassium chloride, sodium ascorbate, ascorbic acid). Other patients typically got Picoprep (sodium picosulfate, magnesium oxide, citric acid). Movicol (polyethylene glycol 3350, potassium chloride, sodium bicarbonate, sodium chloride) was given as standard preparation to patients with reduced kidney function (eGFR < 30 mL/min/1.73 m ^2^ ).

Colonoscopy was only considered complete and included in the study if the cecum/ileocolic anastomosis was intubated. Bowel preparation was evaluated by the endoscopist using a modified version of the Aronchick scale 17 ; graded as good, suboptimal, or poor – indication for new colonoscopy. The AI system was not turned on until the cecum or the ileocolic anastomosis was reached.

Characteristics of polyps detected during the colonoscopy were reported. These included polyp position (cecum, ascending colon, transverse colon, descending colon, sigmoid colon, or rectum), polyp size, and polyp morphology (polypoid or non-polypoid). All polyps were removed, except for some small hyperplastic polyps if the endoscopist believed they were without clinical significance or decided that there was no indication for removing them. Removed adenomas were histologically confirmed.

### Outcome measures

The primary outcome was the ADR in the CADe and control groups. ADR was defined as the proportion of patients with at least one histologically confirmed adenoma.

Second, we performed subgroup analyses on ADR in the two endoscopist experience populations and colonoscopies conducted before and after noon, as well as a subset analysis on the screening population. We also looked at potential differences between the four centers.

Additional secondary outcomes were non-neoplastic resection rate (NNRR), polyp detection rate (PDR), adenomas per colonoscopy (APC), adenomas per positive colonoscopy (APPC), non-adenomas per colonoscopy (NAPC), polyp size, polyp type and localization of polyps, and procedure duration. NNRR was defined as the proportion of patients with at least one histologically confirmed non-neoplastic lesion and no adenomas. APC was defined as the number of detected adenomas divided by the total number of colonoscopies in the study population. APPC was defined as the number of detected adenomas divided by the number of colonoscopies with at least one histologically confirmed adenoma. Sessile serrate lesions with dysplasia and small cancerous polyps were considered adenomas when calculating ADR, APC, APPC, NNRR, and NAPC outcomes.

### Statistical methodology

#### Sample size

ADR in the screening population in Denmark was between 42% and 73% in 2020 18 . We calculated sample size based on ADR for colonoscopies with and without AI assistance in a population we assumed to be similar to ours when comparing the baseline ADR in our population with their baseline ADR 14 .

After reviewing the available literature using multicenter trials to assess ADR in AI-assisted versus non-AI-assisted colonoscopies, we found an increase in ADR from 6% to 15.2% in absolute percentages 8 11 . Of those studies, the most similar in design and population found an increase in ADR of 14.4% 14 . Because there is no consensus on minimally detected effect (MDE) in ADR increase, we used the increase of 14.4 % as MDE because it was also within the range of ADR increase in the literature.

We calculated a minimum sample size of 316 per study group, based on a power of 90%, and a two-tailed test, with a significance level of 0.05 and MDE of 14.4% 14 . We chose a safety margin of 25% to ensure that we would not be underpowered due to possible dropouts and the increased variability introduced by clustering and the relatively small cluster sizes 19 . This adjustment aimed to preserve the study’s statistical power.

### Analyses

Per-protocol analysis outcomes were reported in tables in the results section. Descriptive statistics describing patient characteristics were presented in one table and the primary and secondary outcomes were presented in another. Continuous variables were reported as mean values. Categorical variables were presented as counts and percentages.

We used the Fisher´s exact test to examine the association between categorical variables in the two study groups. Using the Fisher’s exact despite relatively large sample size was done to avoid overestimation of statistical significance.

In the case of continuous variables, we used the Mann-Whitney U-test to test for statistical significance because the included continuous variables were assumed to not be normally distributed.

When applicable for calculated outcome percentages in the AI-assisted colonoscopy and control groups, 95% confidence intervals (CIs) were reported. Results were considered statistically significant if the two-tailed *P* < 0.05.

To adjust for potential confounders in the relationship between the intervention and the outcome, we performed multiple logistic regression with backwards elimination. First, a baseline logistic regression was conducted without any covariates to establish an initial odds ratio (OR). Subsequently, we performed multivariable logistic regression with all potential confounder eliminating one at a time, evaluating whether the OR for the intervention changed by more than 10% compared with the baseline model. Variables that resulted in a substantial change (> 10%) in the OR were retained in the final model.

All statistical analyses were performed using R software version 4.2.1 (2022–06–23).

Planning, execution, and reporting of results from this study was done in accordance with the Consolidated Standards of Reporting Trails (CONSORT) guideline for randomized clinical trials 20 .

## Results

### Study population and baseline data

**Fig. 3:**
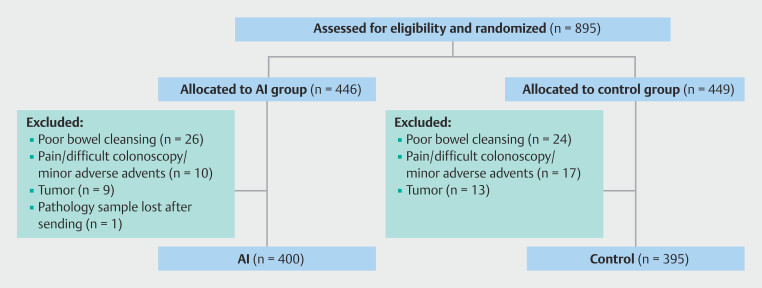
Study flow chart.

Between October 2022 and March 2023, 895 patients were deemed eligible for the study. After excluding 100 patients, 795 patients were included in the final analysis. Among these, 400 were assigned to the AI group, and 395 were assigned to the control group ( Fig. 3 ). In the AI group, one patient had insufficient histopathology data and consequently was excluded from analyses where polyp histology was necessary including the ADR, NNRR, APC, APPC, and NAPC.

Table 1 summarizes baseline characteristics. Experts performed 646 colonoscopies, and non-experts performed 149. Of the included patients, 55.8% were male in the AI group and 50.6% in the control group. Mean age was 64 years in the AI group and 63 years in the control group. Most patients were American Society of Anesthesologists score 2; 74.0% in the AI group and 75.4% in the control group. The most frequent indication for colonoscopy was a positive FIT test; 40.0% in the AI group and 40.5% in the control.

**Table:** **Table 1** Baseline characteristics.

**Variable**	**CADe (400 patients)**	**Control (395 patients)**
**Age (years) mean (± SD)**	63.96 (± 11.12)	63.35 (± 11.14)
**Sex n (%)**		
Female	177 (44.3)	195 (49.4)
Male	223 (55.8)	200 (50.6)
**ASA score n (%)**		
1	31 (7.8)	26 (6.6)
2	296 (74.0)	298 (75.4)
3	73 (18.3)	71 (18.0)
**Comorbidity n (%)**		
Cardiac	73 (18.3)	74 (18.7)
Hypertension	146 (36.5)	135 (34.2)
Obesity	17 (4.3)	20 (5.1)
Pulmonary	51 (12.8)	51 (12.9)
Renal	6 (1.5)	8 (2.0)
Diabetes	48 (12.0)	54 (13.7)
Neurologic	7 (1.8)	11 (2.8)
Other	20 (5.0)	41 (10.4)
No comorbidity	181 (45.3)	156 (39.5)
**Previous intraabdominal surgery n (%)**		
Yes	123 (30.8)	124 (31.4)
No	191 (47.8)	189 (47.8)
Missing	86 (21.5)	82 (20.8)
**Reason for referral n (%)**		
Diagnostic colonoscopy (cancer suspected)	68 (17.0)	108 (27.3)
Diagnostic colonosocopy (benign disease suspected)	49 (12.3)	39 (9.9)
Screening colonoscopy (positive FIT test)	160 (40.0)	160 (40.5)
Post polypectomy surveillance	89 (22.3)	56 (14.2)
Post CRC surgery control	13 (3.3)	14 (3.5)
HNPCC control	10 (2.5)	10 (2.5)
Diverticulitis follow-up	11 (2.8)	8 (2.0)
**Quality of bowel cleansing n (%)**		
Good	227 (56.8)	216 (54.7)
Suboptimal	51 (12.8)	51 (12.9)
Missing	122 (30.5)	128 (32.4)
**Sedation n (%)**		
Yes	218 (54.5)	240 (60.8)
Midazolam + fentanyl	161 (40.3)	180 (45.6)
Midazolam + sufentanil	42 (10.5)	51 (12.9)
Midazolam	0	1
Fentanyl	4	1
Sufentanil	1	1
Missing	10	6
No	182 (45.5)	155 (39.2)
**Diverticulosis**		
Yes	193 (48.3)	158 (40.0)
No	207 (51.8)	237 (60.0)
**Endoscopist experience n (%)**		
> 1000 procedures	333 (83.3)	313 (79.2)
≤ 1000 procedures	67 (16.8)	82 (20.8)
**Supervised**		
Yes	14 (20.9)	21 (25.6)
No	53 (79.1)	61 (74.4)
**Profession n (%)**		
Medical doctor	112 (28.0)	126 (31.9)
Nurse	288 (72.0)	269 (68.1)
**Time of the day n (%)**		
≤ 12 noon	261 (65.3)	259 (65.6)
> 12 noon	139 (34.8)	136 (34.4)
ADR, adenoma detection rate; ASA, American Society of Anesthesiologists; CADe, computer-aided detection; CRC, colorectal cancer; FIT, fetal immunochemical test; HNPCC, hereditary nonpolyposis colorectal cancer; SD, standard deviation.		

None of the variables assessed in the multiple logistic regression, including sex, ASA score, comorbidity, and procedure duration, resulted in a change of more than 10% in the OR. Thus, no evidence of confounding was identified in this analysis.

### Polyps

A total of 1404 polyps were removed and sent for histopathological examination. Of the total amount, 987 polyps were histologically verified adenomas or sessile serrate lesions with dysplasia.

### Primary outcome

Compared with the control group, the AI group had a statistically significant higher ADR (59.1%, 95% CI 54.1–64.0 vs. 46.6%, 95% CI 41.6–51.6, *P* < 0.001).

## Secondary outcomes

AI assistance increased ADR significantly in the screening population compared with patients in the screening population without AI (74.4%, 95% CI 66.8–80.8 vs. 58.1%, 95% CI 50.1–65.8, *P* = 0.003).

### Subgroup analyses

The difference in ADR in the expert group was similar to what was seen in the overall population with a significant increase in ADR (59.9%, 95% CI 54.4–65.2 vs. 47.3%, 95% CI 41.7–53.0, *P* = 0.002). No significant increase was shown in the non-expert group (55.2%, 95% CI 42.6–67.2 vs. 43.9%, 95% CI 33.1–55.3, *P* = 0.190).

ADR was higher in the AI group compared with the control group both before and after 12 noon. Before 12 noon, the ADR was 57.7%, 95% CI 51.4–63.7 in the AI group versus 46.7%, 95% CI 40.5–53.0 in the control group ( *P* = 0.014). After 12 noon, the ADR was 61.9%, 95% CI 53.2–69.9 in the AI group versus 46.3%, 95% CI 37.8–55.1 in the control group ( *P* = 0.011).

When comparing ADR before versus after noon in the AI group and the control group, there was no significant difference ( *P* = 0.455 vs. *P* = 1.0, respectively).

### Per-patient analysis

PDR was significantly higher in the AI group compared with the control group (69.8 vs. 56.2%, *P* < 0.001). No statistically significant difference in NNRR between the AI and control groups was found (15.1% vs. 17.1%) ( *P* = 0.542). CADe increased APC significantly (1.35 vs. 1.11) ( *P* < 0.001). APPC was 2.28 in the AI group and 2.38 in the control group ( *P* = 0.383). CADe increased NAPC significantly (0.58 vs. 0.47) ( *P* = 0.003).

### Per-polyp analysis

Use of CADe contributed to increased detection of both adenomas (539 in 399 patients in the AI group and 438 in 395 patients in the control group; *P* < 0.001) and non-adenomas (233 in 399 in the AI group and 184 in 395 in the control group) ( *P* = 0.003). Most polyps were found in the sigmoid colon; 200 in the AI group and 164 in the control group ( *P* = 0.990). The second most polyps were found in the ascending colon; 161 in the AI group and 92 in the control group ( *P* = 0.015). There was no significant difference in number of polyps in the other segments ( Table 2 ).

**Table:** **Table 2** Outcomes.

**Outcome**	**CADe**	**Control**	***P* value **
**Total number of polyps removed**	804	631	
Removed later (%)	25	22	
Number of lost polyps	22	9	
**Polyps sent for histopathological examination**	782	622	
Localization* n (%)			
Cecum	65 (8.3)	63 (10.1)	0.840
Ascending colon	161 (20.6)	92 (14.8)	0.015†
Right flexure	30 (3.8)	33 (5.3)	0.475
Transverse colon	132 (16.9)	100 (16.1)	0.160
Left flexure	28 (3.6)	14 (2.3)	0.136
Descending colon	64 (8.2)	62 (10.0)	0.890
Sigmoid colon	200 (25.6)	164 (26.4)	0.990
Rectum	102 (13.0)	94 (15.1)	0.924
**Polyp type n (%)**			
Polypoid	80 (10.2)	64 (10.3)	
Non-polypoid	702 (89.8)	558 (89.7)	
**Size polyps n**			
1–5 mm	623	478	< 0.001†
6–9 mm	84	73	0.223
≥ 10 mm	75	71	0.854
**Size adenomas n**	549	438	
1–5 mm	413	323	< 0.001†
6–9 mm	63	54	0.108
≥ 10 mm	63	61	0.831
**Histopathology**			
Hyperplasia	82 (10.5)	72 (11.6)	
Tubular adenoma low-grade	517 (66.1)	412 (66.2)	
Tubular adenoma high-grade	9 (1.2)	11 (1.8)	
Tubulovillous adenoma low-grade	4 (0.5)	7 (1.1)	
Tubulovillous adenoma high-grade	1 (0.1)	0 (0)	
Sessile serrate lesion without dysplasia	89 (11.4)	67 (10.8)	
Sessile serrate lesion with dysplasia/sessile serrate adenoma	7 (0.9)	7 (1.1)	
Granulation tissue	2 (0.3)	2 (0.3)	
Inflammatory polyp	7 (0.9)	5 (0.8)	
Adenocarcinoma	1 (0.1)	1 (0.2)	
Normal tissue	34 (4.3)	24 (3.9)	
Other (benign)	19 (2.4)	14 (2.3)	
Missing	10 (1.3)	0	
Adenoma	539 (69.8)	438 (70.4)	< 0.001†
Non-adenoma	233 (30.2)	184 (29.6)	0.003†
**ADR in the whole population (%) (95% CI)**	59.1 (54.1–64.0)	46.6 (41.6–51.6)	< 0.001†
**ADR in the screening population (%) (95% CI)**	74.4 (66.8–80.8)	58.1 (50.1–65.8)	0.003†
**ADR in the endoscopist subgroups (%) (95% CI)**			
Expert (n = 645)	59.9 (54.4–65.2)	47.3 (41.7–53.0)	0.002†
Non-expert (n = 149)	55.2 (42.6–67.2)	43.9 (33.1–55.3)	0.190
**ADR during the day (%) (95% CI)**			
≤ 12 noon	57.7 (51.4–63.7)	46.7 (40.5–53.0)	0.014†
> 12 noon	61.9 (53.2–69.9)	46.3 (37.8–55.1)	0.011†
**Polyp detection rate (PDR) (%) (95% CI)**	69.8 (64.9–74.2)	56.2 (51.1–61.1)	< 0.001†
**Non-neoplastic resection rate (NNRR) (%) (95% CI)**	15.1 (11.2–20.0)	17.1 (12.5–22.9)	0.542
**Non-adenomas per colonoscopy (NAPC) mean**	0.58	0.47	0.003†
**Adenomas per colonoscopy (APC) mean**	1.35	1.11	< 0.001†
**Adenomas per positive colonoscopy (APPC) mean**	2.28	2.38	0.383
**Duration of the procedure (minutes)**			
Mean	30.00	30.72	0.535
Median (IQ1-IQ3)	28 (21–37)	27 (20–37)	
*Mann-Whitney U-test to determine the difference in number of polyps per intervention, stratified by bowel segment. †P < 0.05 is considered significant ADR, adenoma detection rate; CADe, computer-aided detection.			

A Mann-Whitney U-test was used to determine if exposure to CADe was statistically significantly associated with number of polyps detected. There was an increased detection of diminuitive polyps (1–5 mm) in the AI group (623 vs. 478) ( *P* < 0.001). No difference in number of small (6–9 mm) (84 vs. 73) ( *P* = 0.223) and large polyps (≥ 10 mm) (75 vs. 71) ( *P* = 0.854) was shown. Adenomas described separately under "per-adenoma analysis" ( Table 2 ).

The most frequent histology of polyps was tubular adenoma with low-grade dysplasia; 66.1% in the AI group, and 66.2% in the control group. Only one of the polyps in each group turned out to be an adenocarcinoma. Non-dysplastic polyps accounted for 30.2% of the removed polyps in the AI group and 29.6% in the control group ( Table 2 ).

### Per-adenoma analysis

There was increased detection of diminuitive adenomas (1–5 mm) in the AI group (413 vs. 323) ( *P* < 0.001), but no difference in the number of small (6–9 mm) (63 vs. 54) and large adenomas (≥ 10 mm) (63 vs. 61).

### Procedure duration

We found no significant difference in procedure duration between the control and AI groups. Mean duration in the AI group was 30.00 minutes, with a median of 28 minutes (Q1-Q3: 21–37). Mean duration in the control group was 30.72 minutes, with a median of 27 minutes (Q1-Q3: 20–37).

## Discussion

ADR is one of the key performance measures for colonoscopy 21 . AI in colonoscopy with real-time detection of adenomas can reduce performance variability, compensating for perceptual errors due to fatigue, distraction, and inaccurate human vision, and increase ADR 22 23 .

In this quasi-randomized controlled trial, use of CADe increased ADR significantly by 12.5%. The increase for expert endoscopists was 12.6 %. No significant increase was shown in the non-expert group, possibly due to small sample size. When looking at the effect CADe has on ADR throughout the day, we showed an increase of 11.0% before noon, and 15.6% after noon.

In two previous European RCTs, use of CADe increased ADR significantly with 14.4% (685 patients) and 15.5% (232 patients) respectively 14 24 . The increase in ADR is comparable to our results. One of the studies showed an increase in median withdrawal time 24 , consistent with earlier results 25 . We measured total duration of the procedures and did not find increased procedure time.

The CRC screening program was implemented in Denmark in 2014. Since then, all citizens between ages 50 and 74 have been offered screening for CRC with a FIT test. In 2022, participation in the CRC screening was 59.8% and compliance with receiving a colonoscopy after a positive FIT test was 90.1%. ADR in this screening population was 58.0% 26 . We wanted to investigate whether CADe could benefit these individuals, and we found that the difference in ADR was even higher in the screening population than in the overall population, with a 16.3% absolute increase. This is interesting because baseline ADR is higher in this population than the overall population, and experienced medical doctors and nurses perform all the colonoscopies. For comparison, results from a multicenter RCT from Italy with 800 patients included within a FIT-based CRC screening program showed a significant increase of 8.3% (53.6% vs. 45.3%) 27 .

Colonoscopy quality is shown to be impaired throughout the day; ADR decreases by 7% after the seventh colonoscopy within a procedure day 28 . Although CADe may compensate for decrease in colonoscopy quality throughout the day, we could not show a decrease in ADR after noon in the control group. One potential explanation is that the maximum number of colonoscopies per room per day in our centers was only seven. Further studies in centers with a higher volume of colonoscopies are needed to evaluate the actual effect of AI assistance on colonoscopies throughout the day.

There was increased detection of diminutive polyps and adenomas in the AI group, but no significant difference in detection of small and large polyps and adenomas. This finding is consistent with results from previous studies in which increased ADR was mainly due to increased detection of diminutive adenomas (≤ 5 mm) 23 . In perspective, 2352 diminutive polyps must be removed to prevent one case of CRC over a 10-year period 29 . Clinical significance of CADe in this setting, therefore, is still questionable. It is also notable that the number of advanced adenomas was low in both groups, with 10 in the AI group and 11 in the control group.

NNRR was calculated to investigate whether use of CADe increases the number of patients where only non-adenomas are resected. We found no difference in NNRR, which means that the increase in PDR is driven by increased ADR and not increased NNRR. Both APC and NAPC increase with use of CADe. In other words, more adenomas and non-adenomas are removed, but the number of patients with only non-adenomas remains the same although the number of patients with adenomas increases. No significant difference in APPC was shown.

It is important to note that a system designed solely to detect and characterize polyps cannot compensate for suboptimal colonoscopy technique. The effectiveness of the CADe-system depends on the quality of mucosal exposure. It has previously been shown that computer-aided quality improvement systems with real-time monitoring of withdrawal speed can improve colonoscopy quality and increase ADR 30 31 . The quality improvement system did improve efficacy of the CADe-system with a further increase in ADR 30 . A quality control system also has been developed with the ability to simultaneously detect polyps, supervise withdrawal, and evaluate bowel preparation which shows promising results with a significant increase in ADR 32 .

The main strength of this study is that it was multicenter with a large sample size, powered to show significant results in some relevant subgroups. Moreover, we investigated a population from the Nordic region with high baseline ADR. We also had very few exclusion criteria, resulting in a diverse study population, which we believe is representative of the segment of the Danish population receiving colonoscopies. One of the study strengths and limitations is that it was quasi-randomized at cluster level. This is an advantage because it makes the randomization feasible and ensures clinician compliance with study inclusion guidelines. One weakness of the study design is potential for selection bias because the quasi-randomized approach lacks full randomization control. This study is also limited by risk of confounding. Although we addressed this by performing a multiple logistic regression analysis and did not identify any significant confounders, the possibility of unmeasured confounding cannot be ruled out.

Another limitation with this study is that it was not powered to detect a significant increase in ADR in the non-expert group or the separate centers. Only a limited number of RCTs are investigating the impact of AI when used by less experienced endoscopists compared with experts, and this needs to be further investigated.

## Conclusions

In this multicenter, quasi-randomized, controlled, crossover trial, use of CADe in colonoscopy increased ADR significantly both before and after noon, without increasing NNRR or procedure duration. CADe also benefited the expert endoscopists and significantly increased ADR in patients with a positive FIT test.
